# Cardiovascular risk factors are major determinants of thrombotic risk in patients with the lupus anticoagulant

**DOI:** 10.1186/s12916-017-0807-7

**Published:** 2017-03-10

**Authors:** Florian Posch, Johanna Gebhart, Jacob H. Rand, Silvia Koder, Peter Quehenberger, Vittorio Pengo, Cihan Ay, Ingrid Pabinger

**Affiliations:** 1 Clinical Division of Haematology and Haemostaseology, Department of Medicine I, Comprehensive Cancer Center Vienna, Medical University of Vienna, Währinger Gürtel 18-20, Vienna, 1090 Austria; 2 Division of Oncology, Department of Internal Medicine, Medical University of Graz, Vienna, Austria; 3 Department of Pathology and Laboratory Medicine, Weill Cornell Medical College, New York, NY USA; 4 Department of Laboratory Medicine, Medical University of Vienna, Vienna, Austria; 5 Division of Cardiac, Thoracic, and Vascular Sciences, University of Padova, Padova, Italy

**Keywords:** Lupus anticoagulant, Antiphospholipid antibody syndrome, Thrombotic risk, Risk stratification model, Second hit, diabetes, aPTT, Smoking, Cardiovascular risk factors

## Abstract

**Background:**

Patients with the lupus anticoagulant (LA) are at an increased risk of thrombotic events, which in turn increase the risk of death. Understanding the determinants of thrombotic risk in patients with LA may pave the way towards targeted thromboprophylaxis. In the Vienna Lupus Anticoagulant and Thrombosis Study (LATS), we systematically evaluate risk factors for thrombotic events in patients with LA.

**Methods:**

We followed 150 patients (mean age: 41.3 years, female gender: *n* = 122 (81.3%), history of thrombosis or pregnancy complications: *n* = 111 (74.0%)), who tested repeatedly positive for LA until development of thrombosis, death, or censoring. The primary endpoint was a composite of arterial or venous thrombotic events (TEs).

**Results:**

During a median follow-up of 9.5 years (range: 12 days–13.6 years) and 1076 person-years, 32 TEs occurred (arterial: *n* = 16, venous: *n* = 16; cumulative 10-year TE incidence: 24.3%). A prolonged lupus-sensitive activated partial thromboplastin time (aPTT-LA) (adjusted subdistribution hazard ratio (SHR) = 2.31, 95% CI: 1.07–-5.02), diabetes (adjusted SHR = 4.39, 95% CI: 1.42–13.57), and active smoking (adjusted SHR = 2.31, 95% CI: 1.14–5.02) emerged as independent risk factors of both arterial and venous thrombotic risk. A risk model that includes a prolonged lupus-sensitive aPTT, smoking, and diabetes enabled stratification of LA patients into subgroups with a low, intermediate, and high risk of thrombosis (5-year TE risk of 9.7% (*n* = 77), 30.9% (*n* = 51), and 56.8% (*n* = 22).

**Conclusions:**

Long-term thrombotic risk in patients with LA is clustered within subjects harboring typical cardiovascular risk factors in addition to a prolonged lupus-sensitive aPTT, whereas patients with none of these risk factors represent a large subgroup with a low risk of thrombosis.

**Electronic supplementary material:**

The online version of this article (doi:10.1186/s12916-017-0807-7) contains supplementary material, which is available to authorized users.

## Background

Autoantibodies directed against phospholipid-binding plasma proteins (also known as antiphospholipid (aPL) antibodies), detected through coagulation assays such as the lupus anticoagulant (LA) and through immunoassays such as anticardiolipin (aCL) or anti-β2-glycoprotein I (aβ2-GPI), confer an increased risk of arterial and venous thrombosis [1]. In vitro, the interference by these autoantibodies with the clotting cascade is reflected in the prolongation of phospholipid-dependent clotting assays, such as the activated partial thromboplastin time (aPTT) [2]. Conversely, in vivo, a complex and still enigmatic interplay of these antibodies with coagulation factors, platelets, and the vessel wall induces a hypercoagulable state that can lead to overt thrombosis and/or adverse pregnancy outcomes [3]. The clinical manifestation of these events in a patient who is persistently positive for one or more of these autoantibodies defines the antiphospholipid syndrome (APS) [4].

Cervera et al. and our group have recently demonstrated that the occurrence of thrombosis in patients with LA is associated with an excessive increase in the risk of death, which in turn leads to a significantly impaired relative survival of these patients in comparison to an age- and sex-matched reference population [5, 6]. While anticoagulation may reduce the risk of thrombosis, it is currently unclear which patient groups are at the highest risk for the development of thrombotic complications [3]. Further, a considerable proportion of patients with LA will not develop thrombosis despite the presence of aPL-associated autoantibodies at high titers [7]. The large uncertainty around the potential determinants of thrombotic risk in LA-positive patients is further aggravated by inconsistent study results on the role of aPL-associated autoantibodies for thrombotic risk stratification, and the rarity of adequately powered prospective studies [1, 7–16]. A robust risk stratification tool for long-term thrombotic outcomes in patients with LA therefore represents an unmet clinical need [3].

Here, we report results from a prospective observational cohort study in which we studied the course of disease in patients with LA over time. By systematically analyzing risk factors for the occurrence of thrombosis, we develop a rational basis for thrombotic risk stratification in patients with LA.

## Methods

### Study design and endpoint

The Vienna Lupus Anticoagulant and Thrombosis Study (LATS) is an ongoing, single-center, biobank-based, prospective observational cohort study enrolling adult patients who repeatedly test positive for LA (two positive tests at least 12 weeks apart) with or without a history of thrombosis or pregnancy complications [5]. The primary endpoint of the study is a composite of symptomatic, objectively confirmed arterial and/or venous thrombosis. Comprehensive details about the design of this study have been reported previously [5] and can be found in Additional file 1: paragraph 1.

### Determination of LA and LA-associated autoantibodies

Blood sample preparation procedures are reported in Additional file 2: paragraph 2. LA was diagnosed according to Scientific and Standardization Committee (SSC)/International Society on Thrombosis and Hemostasis (ISTH) recommendations [17, 18]. A lupus-sensitive activated partial thromboplastin time (PTT-LA, Diagnostica Stago, Asniere-sur-Seine, France) and a diluted Russell’s viper venom time (dRVVT) were used as screening tests. For screening during therapy with vitamin K antagonists (VKAs), only the aPTT was used. Confirmatory tests were performed following the methodology of Wenzel et al. in the case of prolongation of one or both screening tests [19]. As confirmatory assays the StaClot LA (Diagnostica Stago, Asniere-sur-Seine, France) and the dRVVT-LA Confirm (Life Diagnostics, Clarkston, GA, USA) were used. In case of a not definitely positive confirmatory test during the follow-up period, LA was still regarded as positive if the Rosner Index, calculated as 100 × (clotting times of the 1:1 mixture - normal plasma)/patient’s plasma, was higher than 15 [20]. Commercially available indirect solid-phase enzyme immunoassays were used to determine IgG and IgM antibodies against cardiolipin (aCL) and β2-GPI. Between 2001 and September 2005, the Varelisa Cardiolipin test (Pharmacia (Phadia AB), Uppsala, Sweden) was performed semi-automatically with a Tecan Genesis liquid handling system (Tecan Group Ltd., Maennedorf, Switzerland). From October 2005 the Orgentec Cardiolipin and, from October 2006, the Orgentec β2-GPI tests (both from Orgentec, Mainz, Germany) were performed on a fully automated BEP2000 Advance System (Siemens Healthcare Diagnostics, Marburg, Germany). All assays were used following the manufacturers’ instructions. Positivity for aCL IgG and aCL IgM was defined as results >40 IgG phospholipid units (GPL)/IgM phospholipid units (MPL) U/mL for both the Varelisa Cardiolipin and the Orgentec Cardiolipin tests according to the revised Sapporo criteria [4]. Two further aCL cut-offs were analyzed as a sensitivity analysis (see the legends of Tables 1 and 3). For aβ2-GPI IgG and IgM (Orgentec assays), results >8U/mL were regarded as positive (corresponding to the 99th percentile of healthy controls). IgM- and IgG-isotype antibodies against prothrombin and protein Z were measured using commercially available enzyme-linked immunosorbent assay kits from the Zymutest product line (Hyphen Biomed, Neuville-sur-Oise, France). The annexin A5 resistance ratio (A5R) was measured as previously described, expressed as the ratio of in vitro coagulation times with and without annexin A5 [21]. IgG-isotype autoantibodies against domain I of β2-GPI were measured with a chemiluminescent immunoassay (QUANTA Flash/Bioflash, Inova Diagnostics, San Diego, CA, USA) [9].

**Table 1:** Baseline characteristics of the study population

Variable	Number (% missing)	Overall (*n* = 150)	Prior history of thrombosis (*n* = 98)	Without prior history of thrombosis (*n* = 52)	*p* ^a^	No event during follow-up (*n* = 118)	Event during follow-up (*n* = 32)	*p* ^a^
Demographic characteristics								
Age at entry (years)	150 (0.0%)	41.3 [32.3-60.2]	39.2 [29.9-54.7]	48.4 [35.6-62.9]	**0.014**	40.1 [31.8-58.2]	46.0 [32.5-63.8]	0.210
Female gender	150 (0.0%)	122 (81.3%)	82 (83.7%)	40 (76.9%)	0.313	98 (83.1%)	24 (75.0%)	0.300
BMI (kg/m^2^)	147 (2.0%)	25.4 [22.5-29.4]	25.6 [22.4-29.9]	25.1 [22.8-28.9]	0.659	25.0 [22.2-29.3]	27.5 [24.6-30.1]	**0.054**
Clinical history								
Prior history of thrombosis	150 (0.0%)	98 (65.3%)	-	-	-	77 (65.3%)	21 (65.6%)	0.969
-Arterial	150 (0.0%)	21 (14.0%)	-	-	-	15 (12.7%)	6 (18.8%)	0.383
-Venous	150 (0.0%)	84 (56.0%)	-	-	-	68 (57.6%)	16 (50.0%)	0.441
-Both	150 (0.0%)	7 (4.7%)	-	-	-	6 (5.1%)	1 (3.1%)	0.538
Prior history of pregnancy complications^b^	94 (0.0%)	40 (42.6%)	-	-	-	32 (42.7%)	8 (42.1%)	0.965
Established APS	150 (0.0%)	111 (74.0%)	98 (100.0%)	13 (25.0%)	**<0.0001**	89 (75.4%)	22 (68.8%)	0.445
Family history of thrombosis	150 (0.0%)	48 (32.0%)	33 (33.7%)	15 (28.9%)	0.546	40 (33.9%)	8 (25.0%)	0.339
Comorbidities								
Hypertension	150 (0.0%)	44 (29.3%)	26 (26.5%)	18 (34.6%)	0.301	34 (28.8%)	10 (31.3%)	0.788
Diabetes	150 (0.0%)	10 (6.7%)	5 (5.1%)	5 (9.6%)	0.292	4 (3.4%)	6 (18.8%)	**0.002**
Statin exposure	150 (0.0%)	10 (6.7%)	8 (8.2%)	2 (3.9%)	0.313	7 (5.9%)	3 (9.4%)	**0.445**
Autoimmune rheumatic diseases^c^	150 (0.0%)	48 (32.0%)	31 (31.7%)	17 (32.7%)	0.895	39 (33.1%)	9 (28.1%)	0.596
-SLE	150 (0.0%)	29 (19.3%)	20 (20.4%)	9 (17.3%)	0.647	24 (20.3%)	5 (15.6%)	0.549
-LLD	150 (0.0%)	19 (12.7%)	11 (11.2%)	8 (15.4%)	0.466	15 (12.7%)	4 (12.5%)	0.975
Thrombophilia^d^	150 (0.0%)	47 (31.3%)	30 (30.6%)	17 (32.7%)	0.794	35 (29.7%)	12 (37.5%)	0.396
Active smoker at baseline	150 (0.0%)	45 (30.0%)	25 (25.5%)	20 (38.5%)	0.099	30 (25.4%)	15 (46.9%)	**0.019**
Anticoagulation at baseline								
VKA	150 (0.0%)	70 (46.7%)	68 (69.4%)	2 (3.9%)	**<0.0001**	55 (46.6%)	15 (46.9%)	0.979
Low molecular weight heparin (LMWH)	150 (0.0%)	14 (9.3%)	11 (11.2%)	3 (5.8%)	**0.381**	14 (11.9%)	0 (0.0%)	**0.041**
Low dose aspirin (LDA)	150 (0.0%)	37 (24.7%)	24 (24.5%)	13 (25.0%)	0.945	29 (24.6%)	8 (25.0%)	0.961
None	150 (0.0%)	54 (36.0%)	17 (17.4%)	37 (71.2%)	**<0.0001**	43 (36.4%)	11 (34.4%)	0.829
Disease-defining autoantibodies								
aPTT-LA (s)	150 (0.0%)	87.4 [70.1-117.5]	90.2 [72.5-118.1]	82.8 [64.9-115.6]	0.165	85.9 [69.0-109.3]	115.0 [74.4-132.5]	**0.040**
aPTT-LA ratio^e^	150 (0.0%)	2.6 [2.1-3.4]	2.6 [2.1-3.5]	2.4 [1.9-3.9]	0.165	2.5 [2.0-3.2]	3.4 [2.2-3.9]	**0.041**
aβ2-GPI IgM (MPL)	148 (1.3%)	5.6 [2.8-15.7]	5.1 [2.5-14.6]	7.1 [3.0-26.7]	0.197	5.3 [2.6-15.5]	7.1 [3.2-17.1]	0.412
aβ2-GPI IgG (GPL)	149 (0.7%)	9.8 [2.3-50.0]	18.9 [3.1-68.5]	5.5 [1.7-17.0]	**0.005**	9.4 [2.3-48.6]	16.3 [2.6-81.0]	0.522
aCL IgM (MPL)	150 (0.0%)	9.1 [3.7-23.0]	7.6 [3.4-16.2]	13.1 [5.1-32.3]	**0.001**	8.7 [3.5-21.1]	10.6 [5.5-25.5]	0.286
aCL IgG (GPL)	150 (0.0%)	19.1 [6.3-71.7]	35.1 [8.9-99.9]	10.8 [5.5-29.9]	**0.037**	18.4 [6.0-65.9]	26.6 [7.0-111.9]	0.236
LA alone	150 (0.0%)	42 (28.4%)	23 (23.7%)	19 (37.3%)	0.082	34 (29.3%)	8 (25.0%)	0.632
LA + aβ2-GPI (ISTH cut-off)^f^	148 (1.3%)	105 (71.0%)	73 (75.3%)	32 (62.8%)	0.111	81 (69.8%)	24 (75.0%)	0.568
LA + aCL (ISTH cut-off)^f^	150 (0.0%)	67 (44.7%)	51 (52.0%)	16 (30.8%)	**0.013**	50 (42.4%)	17 (53.1%)	0.278
Triple positivity (ISTH cut-off)^f^	148 (1.3%)	64 (43.2%)	49 (50.5%)	15 (29.4%)	**0.014**	47 (40.5%)	17 (53.1%)	0.202
Triple positivity (Padova cut-off)^f^	148 (1.3%)	87 (58.8%)	59 (60.8%)	28 (54.9%)	0.487	66 (56.9%)	21 (65.6%)	0.375
Triple positivity (local cut-off)^f^	148 (1.3%)	93 (62.8%)	64 (66.0%)	29 (56.9%)	0.275	72 (62.1%)	21 (65.6%)	0.712
LA-related autoantibodies								
Domain I abs (CU)	144 (6.0%)	30.3 [2.5-256.0]	64.3 [4.1-498.2]	5.9 [1.5-59.9]	**0.002**	30.8 [3.2-245.3]	24.8 [1.5-265.4]	0.580
Anti-protein Z IgM	144 (4.0%)	6.0 [4.1-9.1]	5.5 [3.8-8.8]	6.3 [4.6-9.6]	0.210	5.9 [4.1-9.3]	6.0 [4.4-8.9]	0.651
Anti-protein Z IgG	144 (4.0%)	3.4 [2.6-5.7]	3.4 [2.6-6.0]	3.3 [2.5-5.6]	0.584	3.4 [2.6-5.6]	3.4 [2.4-6.0]	0.888
Antiprothrombin IgM	146 (2.7%)	5.4 [3.2-10.1]	5.2 [2.6-8.5]	6.5 [3.7-13.3]	**0.031**	5.4 [3.2-9.5]	7.5 [3.6-11.0]	0.331
Antiprothrombin IgG	146 (2.7%)	4.5 [2.1-8.7]	5.2 [1.7-11.2]	4.1 [2.9-6.3]	0.407	4.3 [2.0-8.7]	5.8 [2.3-9.6]	0.514
Annexin A5 ratio (%)	143 (4.7%)	205 [175-250]	193 [164-240]	239 [186-258]	**0.001**	218 [176-254]	186 [164-239]	0.164
Selected laboratory parameters								
C-reactive protein (mg/dL)	148 (1.3%)	0.1 [0.0-0.8]	0.2 [0.0-1.0]	0.1 [0.0-0.4]	0.087	0.1 [0.0-0.7]	0.4 [0.0-0.8]	0.721
Triglycerides (mg/dL)	148 (1.3%)	107 [82-153]	110 [87-155]	103 [70-150]	0.354	106 [82-145]	131 [82-191]	0.197
Cholesterol (mg/dL)	149 (0.7%)	196 [174-225]	191 [168-221]	203 [178-232]	0.239	193 [172-221]	202 [180-233]	0.257
HDL/LDL ratio	144 (4.0%)	0.45 [0.35-0.61]	0.44 [0.35-0.60]	0.49 [0.35-0.67]	0.498	0.46 [0.35-0.63]	0.43 [0.35-0.57]	0.533
Homocysteine (μmol/L)	142 (5.3%)	9.8 [8.4-13.7]	9.7 [8.4-13.8]	10.8 [8.6-13.0]	0.484	9.8 [8.5-12.8]	10.1 [8.2-15.5]	0.519
Fibrinogen (mg/dL)	150 (0.0%)	377 [318-430]	390 [320-456]	357 [297-399]	**0.011**	370 [313-430]	396 [355-442]	0.169

Distribution overall as well as by prior history of thrombosis and prospective thrombotic event status

^a^ *p* values were derived using Wilcoxon’s rank-sum tests (*p* values ≤ 0.05 are reported in bold font)

^b^Pregnancy complications were defined according to Sapporo criteria in the subgroup of 94 females who had at least one documented pregnancy

^c^Autoimmune rheumatic diseases were defined as a composite of systemic lupus erythematosus (SLE) and lupus-like disease (LLD) according to a local panel of rheumatology experts

^d^Hereditary thrombophilia was defined as the presence of at least one of the following factors: (1) heterozygous or homozygous factor V Leiden, (2) deficiency of antithrombin III, (3) deficiency of protein C or protein S, (4) hyperhomocysteinemia, (5) heterozygous or homozygous prothrombin G20210A polymorphism, and (6) high coagulation factor VIII

^e^The aPTT ratio is defined by the ratio of the lupus-sensitive aPTT of a patient divided by the mean of the lupus-sensitive aPTT in healthy controls at our department (mean = 34.09 s, SD = 0.476)

^f^Cut-offs were defined as follows: ISTH cut-off: aCL > 40GPL/MPL U/mL, aβ2-GPI IgG > 8 GPL/MPL U/mL; Padova cut-off (i.e., the cut-off corresponding to the 99th percentile at the laboratory of Prof. Vittorio Pengo (University of Padova, Italy (personal communication)): aCL > 17 GPL/MPL U/mL, aβ2-GPI IgG > 8 GPL/MPL U/mL; local cut-off (i.e., the 99th percentile at the coagulation laboratory of the Medical University of Vienna): aCL ≥ 10 GPL U/mL for aCL IgG, ≥7 for aCL IgM (Orgentec assays), or >15 GPL/MPL U/mL (Varelisa assays)

### Statistical methods

The statistical analysis is described in detail in Additional file 3: paragraph 3. Briefly, median follow-up time was estimated with the reverse Kaplan-Meier estimator [22]. Patients who became LA negative during follow-up (*n* = 11) were censored at the date of the first negative LA test. The cumulative incidence of the primary endpoint was calculated with cumulative incidence estimators according to Marubini and Valsecchi, treating death from any cause as a competing risk [23]. Differences in thrombosis incidence functions between two or more groups were investigated using Gray’s test [24]. The association between potential risk factors and the cumulative incidence of thrombosis was modeled with uni- and multivariable proportional subdistribution hazards models according to Fine and Gray [25]. To increase external generalizability, modeling was also performed using an aPTT ratio, which was defined as the ratio of the lupus-sensitive aPTT of a patient divided by the mean of the lupus-sensitive aPTT in healthy controls at our department (mean = 34.09 s, SD = 0.476). A backward selection algorithm (*p* for exclusion = 0.10) including all four univariable predictors of thrombotic risk with *p* < 0.10 (lupus-sensitive aPTT ratio (dichotomized into a binary variable at the 75th percentile of the distribution (117.5 s)), diabetes, smoking, and aCL IgM antibodies) was applied to construct a multivariable model for the prediction of thrombotic risk [26]. The algorithm selected the three variables diabetes, smoking, and a prolonged lupus-sensitive aPTT, and we constructed an empirical risk stratification rule by assigning 2 points for diabetes, and 1 point for each of the risk factors smoking and a prolonged lupus-sensitive aPTT. These points were chosen because they were consistent with an *additive* effect of the underlying predictor variables on the log hazard scale (further details are reported in Additional file 3: paragraph 3) [27]. Discrimination of the proposed stratification rule was assessed using Harrell’s C statistic, and calibration was explored by comparing the observed and predicted 5- and 10-year cumulative incidences of thrombosis [28]. Finally, in a sensitivity analysis, we assessed the separate association between the three risk stratification variables and the prospective risk of arterial and venous thrombosis (see Additional file 4: Table S1).

## Results

### Analysis at baseline

One hundred and fifty patients were included in the analysis. Patients were predominantly female, and 74.2% had an established diagnosis of APS (Table 1). All patients were positive for LA, and 67 (44.7%), 105 (71.0%), and 64 (43.2%) patients also had above-cut-off antibody levels against cardiolipin (aCL), β2-GPI (aβ2-GPI), or both (“triple positivity”). IgM- and IgG-isotype aCL and aβ2-GPI antibodies were moderately strongly correlated with each other. Some correlations were also observed between elevated levels of these antibodies and (1) higher levels of antibodies against prothrombin and protein Z, (2) a lower annexin A5 anticoagulant ratio, and (3) higher levels of IgG-isotype antibodies against domain I of β2-GPI (Additional file 5: Table S2). A long lupus-sensitive aPTT was significantly correlated with a higher level of IgG-isotype antibodies against domain I of β2-GPI (rho = 0.40, *p* < 0.0001) and a lower prothrombin time (given as percent of normal, rho = –0.27, *p* = 0.0007). The average levels of the lupus-sensitive aPTT and fibrinogen were slightly but non-statistically significantly elevated in VKA users (Additional file 6: Table S3).

### Analysis of anamnestic risk of thrombotic events (TEs) and pregnancy complications

Ninety-eight patients (65.3%) had a history of thrombotic events (TEs) before study inclusion (arterial: *n* =﻿﻿21, venous: *n* = 84, both: *n* = 7). Patients with a history of TE were significantly younger than patients without a history of TE and had a much higher probability of being on oral anticoagulation with VKA (odds ratio (OR) = 56.7, 95% CI: 12.9–248.2, *p* < 0.0001, Table 1). The median levels of IgG-isotype antibodies against aCL and β2-GPI (Table 1) and the odds of being “triple positive” were also significantly higher in patients with prior TE (OR = 2.5, 95% CI: 1.2–5.1, *p* = 0.01). In patients with a prior history of TE, we observed a significantly higher average antibody level against domain I of β2-GPI and a significantly lower annexin A5 anticoagulant ratio. LA-related antibodies against prothrombin and/or protein Z did not appear to consistently differ according to anamnestic thrombosis status. Forty (42.6%) of the 94 female patients who had at least one documented pregnancy had at least one pregnancy complication according to Sapporo criteria. These 40 women had significantly higher levels of IgG-isotype aCL, aβ2-GPI, and domain 1-β2-GPI antibodies and were also more likely to be “triple positive” (Additional file 7: Table S4). Other parameters did not appear to differ between women with and without pregnancy complications.

### Analysis of prospective risk of thrombosis

During a median follow-up of 9.5 years (range: 12 days–13.6 years) and 1076 patient years, 32 patients developed TE (arterial: *n* = 16, venous: *n* = 16). The most frequent type of events were lower extremity deep vein thrombosis (*n* = 6) and pulmonary embolism (*n* = 6) in the venous vasculature, and cerebrovascular incidents (*n* = 9) and myocardial infarction (*n* = 5) in the arterial vasculature. Twenty-one of the 32 events occurred in patients with a prior history of thrombosis (“recurrent thrombosis”), and 11 events occurred in LA-positive patients without a prior history of thrombosis. Data on antithrombotic therapy at the time of thrombosis were available for 31 out of 32 patients (Table 2). Twenty-three (74.2%) of these 31 events occurred while patients were receiving antithrombotic therapy (Table 2). In detail, 14 (45.2%), 4 (12.9%), and 7 (22.6%) of these patients were receiving VKA, low molecular weight heparin, and/or low dose aspirin at the time of thrombosis, respectively. Among the 14 patients receiving VKA, the international normalized ratio (INR) was insufficient (i.e., <2) in 6 patients, within therapeutic range in 5 patients, and unknown in 3 patients. The cumulative 1-, 5-, 10-, and 15-year incidences of TE accounting for competing mortality were 4.0% (95% CI: 1.7–8.1), 13.3% (95% CI: 8.3–19.4), 24.3% (95% CI: 17.0–32.5), and 27.6% (95% CI: 19.3–36.6), respectively (Additional file 8: Figure S1). With 12 patients having died during follow-up without developing TE, death was clearly present as a competing risk in this population. Of the 32 patients who developed thrombosis during follow-up, 2 patients developed a further TE (1x venous thrombotic event (VTE) after VTE, 1x myocardial infarction after cerebrovascular insult).

**Table 2:** Type of thrombotic event and antithrombotic therapy at the time of event

Type of TE	Total *n* (%)	On VKA *n* (%)	On LMWH *n* (%)	On LDA *n* (%)	No AC *n* (%)
All TE	32 (100.0%)	14 (45.2%)	4 (12.9%)	7 (22.6%)	8 (25.8%)
Venous TE	16 (50.0%)	8 (53.3%)	3 (20.0%)	3 (20.0%)	3 (20.0%)
Lower extremity DVT	6 (18.8%)	4 (66.7%)	0 (0.0%)	0 (0.0%)	2 (33.3%)
Isolated PE	6 (18.8%)	2 (40.0%)	1 (20.0%)	2 (40.0%)	1 (20.0%)
Lower extremity DVT + PE	1 (3.1%)	1 (100.0%)	0 (0.0%)	0 (0.0%)	0 (0.0%)
Upper extremity DVT	1 (3.1%)	0 (0.0%)	1 (100.0%)	1 (100.0%)	0 (0.0%)
Renal vein thrombosis	1 (3.1%)	0 (0.0%)	1 (100.0%)	0 (0.0%)	0 (0.0%)
Ocular vein thrombosis	1 (3.1%)	1 (100.0%)	0 (0.0%)	0 (0.0%)	0 (0.0%)
Arterial TE	16 (50.0%)	6 (37.5%)	1 (6.3%)	4 (25.0%)	5 (31.3%)
Stroke	8 (25.0%)	3 (37.5%)	0 (0.0%)	1 (12.5%)	3 (37.5%)
TIA	1 (3.1%)	1 (100.0%)	0 (0.0%)	1 (100.0%)	0 (0.0%)
Myocardial infarction	5 (15.6%)	1 (20.0%)	1 (20.0%)	1 (20.0%)	2 (40.0%)
Peripheral artery TE	2 (6.3%)	1 (50.0%)	0 (0.0%)	1 (50.0%)	0 (0.0%)

*TE* thrombotic events, *DVT* deep vein thrombosis, *PE* pulmonary embolism, *VKA* vitamin K antagonist, *LMWH* low molecular weight heparin, *LDA* low dose aspirin, *AC* anticoagulation

In a *univariable* competing risk analysis, diabetes (subdistribution hazard ratio (SHR) = 5.18, 95% CI: 1.87–14.31, *p* = 0.002), active smoking (SHR = 2.11, 95% CI: 1.06–4.20, *p* = 0.034), and a prolonged lupus-sensitive aPTT (SHR per 10 seconds increase = 1.10, 1.00–1.21, *p* = 0.044) were univariably associated with a higher risk of TE (Table 3). In detail, the 10-year cumulative risk of TE was 60.0% in patients who were diabetic at baseline, as compared to 21.6% in non-diabetic patients (Gray’s test *p* = 0.002). The 10-year thrombotic risk was estimated at 36.5% in active smokers, as compared to 18.8% in ex- or never-smokers (*p* = 0.022). In patients with a lupus-sensitive aPTT > or ≤ the 75th percentile of its distribution (cut-off at 117.5 s (or for the aPTT ratio at 3.4 multiples of the median in healthy individuals)), we observed 10-year thrombotic risks of 43.7% and 17.6%, respectively (*p* = 0.004, Additional file 9: Figure S2A–C). A borderline significant association was observed between an increased baseline IgM-isotype aCL antibody level and a higher risk of TE risk (SHR = 1.30, 95% CI: 0.98–1.74, *p* = 0.068). Risk of TE was comparable between patients with established APS and LA-positive-only patients (SHR = 0.75, 95% CI: 0.36–1.58, *p* = 0.448), as well as between patients with or without a prior history of thrombosis (SHR = 0.94, 95% CI: 0.45–1.95, *p* = 0.865). Oral anticoagulation with a VKA at baseline was not associated with prospective thrombotic risk (SHR = 0.93, 95% CI: 0.47–1.86, *p* = 0.839, Additional file 10: Figure S3A). Antibodies against domain I of β2-GPI also did not emerge to be associated with prospective risk of thrombosis in the univariable analysis (SHR per 1000 CU increase = 0.93, 95% CI: 0.65–1.34, *p* = 0.711), and this result prevailed when analyzing the subgroups of patients (1) with and without a prior history of thrombosis (*p* for interaction = 0.323) and (2) younger or older than 50 years at study entry (*p* for interaction = 0.514). In a *multivariable* analysis, we adjusted the results for diabetes, smoking, and a prolonged lupus-sensitive aPTT ratio (Table 3, i.e., the variables that were selected below). The joint multivariable association between diabetes, smoking, the prolonged lupus-sensitive aPTT ratio and a higher risk of thrombosis prevailed upon inclusion of all reported variables. This also held true when adjusting for oral anticoagulation at baseline (Additional file 11: Table S5). None of the studied variables was significantly associated with thrombotic risk after adjusting for these three variables. However, a weak multivariable association between exposure to statins and a higher risk of thrombosis was observed. In a sensitivity analysis by event type, diabetes and smoking appeared to contribute prognostic information towards arterial events, and the prolonged lupus-sensitive aPTT towards venous events (Additional file 4: Table S1). Further sensitivity analyses by event type did not identify signals for associations between other studied variables and the risk of arterial and or venous events (not shown).

**Table 3:** Baseline predictors of thrombotic risk in patients with LA: univariable and multivariable analyses

	Univariable analysis			Multivariable analysis		
	SHR	95% CI	*p*	SHR	95% CI	*p*
Demographic characteristics						
Age at entry (per 5 years increase)	1.06	0.97-1.17	0.193	1.07	0.99-1.04	0.174
Female gender	0.73	0.34-1.59	0.433	0.92	0.38-2.23	0.859
BMI (per 5 kg/m^2^ increase)	1.22	0.94-1.58	0.131	1.30	0.94-1.80	0.112
Clinical history						
Prior history of thrombosis	0.94	0.45-1.95	0.865	1.40	0.60-3.27	0.436
-Arterial	1.31	0.55-3.12	0.547	1.08	0.42-2.79	0.872
-Venous	0.73	0.37-1.46	0.374	1.06	0.51-2.20	0.870
-Arterial and venous	0.53	0.09-3.23	0.491	0.38	0.04-3.79	0.409
Prior history of pregnancy complicationsa	0.97	0.39-2.40	0.954	0.53	0.19-1.48	0.225
APS	0.75	0.36-1.58	0.448	0.89	0.39-2.00	0.772
Family history of thrombosis	0.70	0.31-1.57	0.390	0.86	0.37-2.00	0.719
Oral anticoagulation at baseline (VKA)	0.93	0.47-1.86	0.839	1.16	0.56-2.39	0.695
Comorbidities						
Hypertension	1.28	0.60-2.75	0.525	0.79	0.25-2.53	0.697
Diabetes	5.18	1.87-14.31	**0.002**	N/A	N/A	N/A
Statin exposure	2.64	0.71-9.82	0.147	3.34	0.94-11.89	0.063
Autoimmune rheumatic diseases^b^	0.81	0.38-1.74	0.594	0.70	0.31-1.60	0.404
Hereditary thrombophilia^c^	1.22	0.60-2.46	0.586	1.23	0.59-2.55	0.580
Active smoker at baseline	2.11	1.06-4.20	**0.034**	N/A	N/A	N/A
Disease-defining autoantibodies						
aPTT-LA (per 10-s increase)	1.10	1.00-1.21	**0.044**	N/A	N/A	N/A
aPTT ratio (per 1 multiple of the mean)	1.39	1.01-1.93	**0.044**	N/A	N/A	N/A
aPTT or aPTT ratio >75th percentile^d^	2.65	1.32-5.31	**0.006**	N/A	N/A	N/A
aβ2-GPI IgM (per 1 logMPL increase)	1.12	0.87-1.46	0.377	1.00	0.75-1.33	0.993
aβ2-GPI IgG (per 1 logGPL increase)	1.03	0.85-1.25	0.778	1.04	0.84-1.28	0.730
aCL IgM (per 1 logMPL increase)	1.30	0.98-1.74	0.068	1.08	0.76-1.53	0.665
aCL IgG (per 1 logGPL increase)	1.10	0.87-1.39	0.436	1.03	0.80-1.33	0.804
LA alone	0.77	0.35-1.71	0.528	0.91	0.41-2.02	0.825
LA + aβ2-GPI	1.33	0.60-2.95	0.476	1.14	0.51-2.53	0.746
LA + aCL	1.42	0.71-2.82	0.323	1.16	0.56-2.42	0.684
Triple positivity (ISTH cut-off)^e^	1.53	0.77-3.04	0.226	1.22	0.58-2.54	0.598
Triple positivity (Padova cut-off)^e^	1.37	0.67-2.84	0.390	1.00	0.45-2.23	0.998
Triple positivity (local cut-off)^e^	1.21	0.59-2.51	0.603	0.91	0.42-1.99	0.812
LA-related autoantibodies						
Domain I β2-GPI (per 1000 CU increase)	0.93	0.65-1.34	0.711	0.82	0.43-1.56	0.546
Anti-protein Z IgM (per 10 units increase)	1.00	0.58-1.72	0.999	0.75	0.43-1.30	0.301
Anti-protein Z IgG (per 10 units increase)	1.01	0.74-1.38	0.931	0.90	0.67-1.20	0.457
Antiprothrombin IgM	0.98	0.87-1.10	0.747	0.98	0.94-1.01	0.163
Antiprothrombin IgG	0.99	0.96-1.01	0.373	1.00	0.99-1.00	0.340
Annexin A5 ratio (per 50% increase)	0.83	0.56-1.23	0.347	0.92	0.55-1.53	0.734
Selected laboratory parameters						
C-reactive protein (per 5 mg/dL increase)	1.04	0.68-1.57	0.863	0.95	0.63-1.42	0.792
Triglycerides (per 100 mg/dL increase)^f^	1.32	0.86-2.01	0.201	1.19	0.96-1.47	0.121
Cholesterol (per 100 mg/dL increase)	1.22	0.58-2.58	0.604	1.31	0.59-2.92	0.513
HDL/LDL ratio (per 1 unit increase)	0.99	0.82-1.20	0.937	1.04	0.89-1.21	0.601
Homocysteine (per 5 μmol/L increase)^f^	1.25	0.81-1.93	0.316	1.03	0.94-1.12	0.571
Fibrinogen (per 100 mg/dL increase)	1.15	0.80-1.66	0.440	1.23	0.86-1.77	0.253

All presented results are derived from uni- and multivariable Fine and Gray proportional subdistribution hazards regression models (*p* values ≤ 0.05 are reported in bold font). In multivariable analysis, estimates are adjusted for the baseline variables diabetes, smoking, and a prolonged lupus-sensitive aPTT ratio

^a–f^Defined as in the legend of Table 1

^f^Both the triglyceride level and the homocysteine level were univariably associated with a higher risk of thrombosis; however, one outlier was present in each of these variables, and the association between these variables and thrombotic risk disappeared after exclusion of these outliers. The reported subhazard ratios exclude these outliers

*SHR* subdistribution hazard ratio, *95%CI* 95% confidence interval, *p* Wald test *p* value, *VKA* vitamin K antagonist, *N/A* not applicable

### Thrombosis risk stratification in patients with LA

**Fig. 1:**
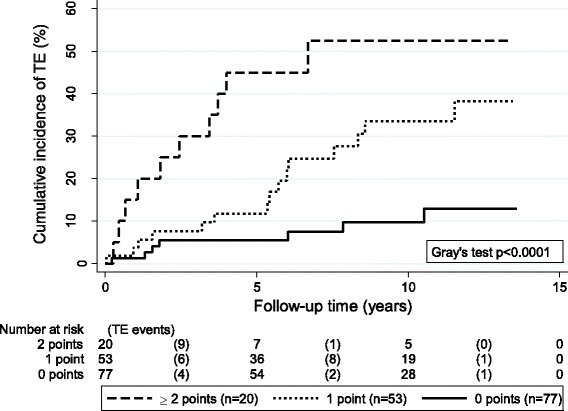
Cumulative incidence of thrombotic risk according to the proposed empirical risk stratification rule. 1 point is assigned for either the baseline presence of active smoking or a prolonged lupus-sensitive aPTT ratio, and 2 points are assigned for the baseline presence of diabetes. *TE* thromboembolic events

A backward selection algorithm included diabetes, smoking, and a prolonged lupus-sensitive aPTT ratio (binary specification) into a model for thrombotic risk stratification for patients with LA (Model 1, Table 4). According to the relative contribution of these variables to the model, 2 points were assigned for diabetes, and 1 point each for smoking and a prolonged lupus-sensitive aPTT (Models 2 and 3, further sensitivity analyses for point assignment are reported in Additional file 12: paragraph 4). The three-category point-based model (Model 3) could stratify patients into subgroups with a very low and very high risk of thrombosis (10-year risk of TE in patients with 0 (*n* = 77), 1 (*n* = 51), or ≥2 (*n* = 22) points: 9.7%, 30.9%, and 56.8%, respectively; Fig. 1). Internal validation procedures showed a strong discrimination according to this point-based rule (Harrell’s C: 0.72), and calibration was excellent for 10-year thrombotic risk and moderate for 5-year thrombotic risk (Additional file 13: Figure S4).

**Table 4:** Multivariable models for thrombotic risk in patients with LA

Models	SHR	95% CI	*p*	Log(SHR)	Assigned point
Model 1					
Diabetes	3.97	1.29-12.19	0.016	1.38	2
Active smoking	2.42	1.15-5.06	0.019	0.88	1
Prolonged aPTT-LA ratio^a^	2.28	1.04-4.99	0.039	0.82	1
Model 2					
0 point (*n* = 77 (51.3%))	Ref.	Ref.	Ref.	-	-
1 point (*n* = 51 (34.0%))	2.84	1.14-7.02	0.024	-	-
2 points (*n* = 15 (10.0%))	8.56	2.91-25.17	<0.0001	-	-
3 points (*n* = 7 (4.7%))	8.45	2.21-32.35	0.002	-	-
Model 3					
0 point (*n* = 77 (51.3%))	Ref.	Ref.	Ref.	-	-
1 point (*n* = 51 (34.0%))	2.84	1.14-7.02	0.024	-	-
≥ 2 points (*n* = 22 (14.7%))	8.53	3.19-22.78	<0.0001	-	-

Model 1 is a multivariable model including the three variables as binary specifications (a prolonged lupus-sensitive aPTT was defined as being above the 75th percentile (Q3) of this variable’s distribution (cut-off: 117.5 s)). Model 2 is a multivariable model based on the points that were assigned for the relative contribution of the individual variables (as represented by the log (subdistribution hazard ratios) in Model 1). In Model 3, the two highest risk categories were combined in a post hoc fashion, because the coefficients showed a similar relative risk for 2 and 3 points. Model 3 is the final product of our prediction model-building strategy, and observed risks according to this point-based rule are shown in Fig. 1

*SHR* subdistribution hazard ratio, *95%CI* 95% confidence interval, *p* Wald test *p* value, *log(SHR)* natural logarithm of the SHR, *Ref* reference category

^a^Prolonged aPTT ratio defined by a prolongation above the 75th percentile of this variable’s distribution (this corresponds to cut-off at 117.5 s (or for the aPTT ratio at 3.4 multiples of the median in healthy individuals))

## Discussion

In this prospective study on patients with persistently positive LA with or without established APS, we observed a high incidence of thrombotic complications. A systematic analysis of risk factors for the occurrence of thrombosis showed that thrombotic risk was highly clustered within subjects who had (1) typical cardiovascular risk factors, such as diabetes or active smoking, and (2) a very prolonged lupus-sensitive aPTT. While disease-defining autoantibodies such as aCL or aβ2-GPI were associated with anamnestic thrombotic risk, these variables appeared to harbor very limited prognostic information on *prospective*, long-term thrombotic outcomes over a median follow-up period of nearly 10 years. Notably, a simple empirical risk stratification rule consisting of two clinical and one laboratory variable allowed us to stratify our patients into subgroups with a very high and very low risk of thrombosis.

Two-thirds of the LA-positive patients in this study had a history of at least one thrombotic event, and venous events were the predominant anamnestic event type. Two-thirds of the patients with a history of thrombosis were on oral anticoagulants at baseline, which reflects the decision of individual physicians to anticoagulate these patients due to a high perceived risk of recurrent thrombosis. In the baseline analysis, we found univariable associations between *some* aPL-associated autoantibodies and the *anamnestic* risk of thrombosis. Specifically, we could observe that the presence of antibodies against domain I of β2-GPI and “triple positivity” were more frequent in patients who had a history of thrombosis. These results confirm and validate several previous reports that have implicated these antibodies in the pathogenesis of APS [9, 10, 12, 13, 29, 30], but they also add to the growing body of evidence that shows inconsistent associations between aPL-associated autoantibodies and anamnestic thrombotic risk [3, 16]. Among non-canonical aPL-associated antibodies [11], such as antibodies against protein Z or prothrombin, we only observed a weak association between higher IgM-isotype antibodies against protein Z and a higher risk of anamnestic thrombosis. Collectively, these findings do not support the concept that antibodies against protein Z or prothrombin correctly identify LA positive patients with a *history* of thrombosis.

After a median follow-up interval of nearly 10 years and more than 1000 person-years spent at risk of thrombosis and death, we observed a high incidence of thrombotic complications despite the fact that the majority of patients with a history of thrombosis were on anticoagulation therapy. Importantly, approximately three out of four thrombotic events occurred while patients were receiving antithrombotic medication with VKA, LMWH, and/or LDA, suggesting that episodes of hypercoagulability in LA-positive patients can induce overt thrombosis despite antithrombotic therapy. The 10-year cumulative TE incidence of 24.3% compares well to the thrombotic risk observed in the WAPS study [31] and the very large Euro-Phospholipid Project registry [6], but was lower than the value in the recently published Piedmont cohort study [32] and higher than that in the recently published study by Pengo et al. [30]. Further, the relative frequencies of arterial and venous events as well as the proportion of patients receiving anticoagulation while developing an event were very similar to the results of the Euro-Phospholipid study [6].

In our study, we were surprised to observe that none of the studied aPL-associated autoantibodies were consistently associated with the risk of developing thrombosis. This held true also for “triple positivity.” While triple positivity has been shown to be a significant thrombotic risk factor in the WAPS study [7], several more recent prospective cohorts have also observed a lack of association between triple positivity or other LA-related autoantibodies and thrombosis [30, 32, 33]. Among the studied antibodies, IgG-isotype antibodies against domain 1 of β2-GPI showed the strongest and most consistent associations with both anamnestic risk of thrombosis and pregnancy complications. However, also with this specific antibody we did not observe an association with prospective thrombotic risk in the overall cohort or when performing subgroup analyses in patients with and without a prior history of thrombosis. One reason for the absence of an association between LA-related autoantibodies and prospective thrombotic risk in our cohort could be that our study population was highly enriched with “high-risk” LA-positive patients, as this was our inclusion criteria and as LA is known to be the strongest laboratory predictor of thrombosis to date in APS patients [3, 29]. However, considering the accumulating prospective evidence about a lack of association between these antibodies and prospective thrombotic risk [3], our results support the suggestion that LA-related autoantibodies may be much more relevant for making the diagnosis of APS, rather than for making a prognostic statement about future thrombotic risk in these patients.

Interestingly, we found very strong associations between the presence of typical cardiovascular risk factors at baseline, such as diabetes and smoking, with an excessively increased risk of thrombosis in our cohort. Further, we have observed a weak association between exposure to statins and a higher risk of thrombosis. This is consistent with the recently published results from the Piedmont cohort study, which found a tenfold increased thrombotic risk in LA patients with diabetes [32]. Further, a risk score for *anamnestic* thrombotic risk in patients with SLE, the Global Anti-Phospholipid Syndrome Score (GAPSS), includes two general cardiovascular risk factors: hypertension and hyperlipidemia [34]. Both hypertension and dyslipidemia were strongly correlated with diabetes in our study. We can derive two hypotheses from these findings. First, diabetes and smoking are modifiable risk factors. Although prospective clinical trial data are absent, we can speculate that smoking cessation efforts and interventions to control blood glucose levels and improve metabolic function may reduce the risk of TE in LA-positive patients. Second, preclinical evidence from animal models has demonstrated that the presence of LA-related autoantibodies alone is insufficient for causing overt thrombosis, and a “second hit” such as inflammation or infection may be necessary to transform the prothrombotic potential induced by LA-related autoantibodies into overt thrombosis [35]. Our data support the concept that diabetes and active smoking may represent two factors for this “second-hit phenomenon” and that the adverse cardiovascular consequences of diabetes and smoking may lead to a deleterious thrombotic risk increase in patients with LA.

Interestingly, a prolongation of a lupus-sensitive aPTT (PTT-LA, Diagnostica Stago, Asniere-sur-Seine, France), which is used as a screening tool for making the diagnosis of LA at our center, was also strongly associated with a higher prospective TE risk, and this association prevailed after controlling for oral anticoagulation and other risk factors including diabetes and smoking. We hypothesize that the association between this laboratory variable and thrombosis risk is likely not causal. Rather, a prolonged lupus-sensitive aPTT may represent a proxy variable for a more aggressive disease phenotype, leading to a stronger polyclonal autoantibody production and thus a stronger in vitro inhibition of the coagulation cascade. This hypothesis is supported by previous case-control studies that have implicated the length of a lupus-sensitive aPTT with thrombosis in LA [13, 36, 37].

In a sensitivity analysis estimating the risk of arterial and venous prospective events separately, we could observe that diabetes and smoking appeared to be more relevant for predicting arterial events, whereas the prolonged lupus-sensitive aPTT was more associated with the occurrence of venous events. Although this analysis has very low power, it illustrates a differential pathobiology of these three risk factors.

Based on our univariable findings and a prespecified model-building algorithm, we identified a simple empirical risk stratification rule including the variables diabetes, smoking, and a prolonged lupus-sensitive aPTT ratio which could discriminate our patients into subgroups with a very high and very low risk of thrombosis. Internal validation procedures showed that this risk stratification rule featured a high discriminative performance and was well calibrated for prediction of 10-year thrombotic risk. Some miscalibration was observed for 1- and 5-year thrombotic risk prediction in patients with 1 or ≥2 points, where the rule somewhat over- and underpredicted the observed thrombotic risk. While this rule still has to be validated in an external cohort, its most promising feature is that it could identify a very large subgroup of patients representing 50% of our patient population who had a very low risk of thrombosis with the current management strategy, namely treating patients with a history of thrombosis with oral anticoagulation. Clinically, an intensification of antithrombotic therapy will likely have a poor benefit-risk ratio in this large subgroup. Conversely, we identified a smaller subgroup representing about 15% of our population who experienced an excessive thrombotic risk.

### Limitations and future research

Although this study represents one of the very few prospectively executed studies with stringent inclusion criteria in the field of APS research, several limitations have to be mentioned. First, this study includes patients who tested repeatedly positive for LA with and without a history of thrombosis and included subgroups of patients with other potentially relevant factors, such as concomitant autoimmune rheumatic diseases. Consequently, our results are not directly generalizable to other patient groups included in the wide and heterogenic spectrum of APS, such as patients with SLE [34, 38] or patients who are positive for aCL or aβ2-GPI but not LA [39]. Second, our present analysis cannot provide a valid estimate for the potential benefit of anticoagulation in LA-positive patients. In this observational study, patients who were anticoagulated at baseline had a similar risk of prospective thrombosis as patients who were not anticoagulated. Of course, this must not be interpreted as an absence of efficacy, because reverse causality has likely confounded this observational result. Indeed, patients with a history of thrombosis had a substantially higher probability of being on oral anticoagulation, so anticoagulation may rather reflect the decision of individual physicians to anticoagulate those patients with the highest perceived risk of thrombosis. Other prospective investigators in the field have also faced this problem [32]. Third, because prospective data in LA-positive patients are scarce, we could not yet validate our empirical risk stratification rule in an external cohort. Fourth, our suggested risk stratification rule has some loss of information as compared to the full multivariable model, which is attributable to “rounding” of regression coefficients to a point-based system. In detail, the relative contribution of diabetes towards thrombotic risk, as compared to smoking status and the lupus-sensitive aPTT, would have led to an uneven point score of 1.6 for diabetes, which was rounded to 2. Nevertheless, we would like to mention that our cohort features several strengths, including a stringent prospective design with a long follow-up, a small drop-out rate, and the time-dependent censoring of patients who became LA negative over time.

## Conclusions

We conclude that established risk factors for vascular events in the general population also turned out to be relevant in patients with LA. This is consistent with the hypothesis that these risk factors represent the necessary “second hit” for eliciting thrombosis in patients with LA. Moreover, a very long lupus-sensitive aPTT was predictive for the occurrence of TE in these patients as well. These associations were independent of anticoagulation. Disease-defining antibodies, such as those against cardiolipin or β2-GPI (including those against domain I), showed a strong association with anamnestic risk of thrombosis; however, they did not predict the future occurrence of TE in this LA-positive patient population. In conclusion, our results suggest that, above standard anticoagulation, interventions to control and improve metabolic status and smoking habits might influence the rates of future TE in patients with known persistent LA. A simple empirical risk stratification rule can identify a very large subgroup of LA-positive patients with a very low prospective risk of thrombosis.

## Additional files

Additional file 1: Paragraph 1. Study design and endpoint. (DOCX 85 kb)
Additional file 2: Paragraph 2. Sample preparation. (DOCX 60 kb)
Additional file 3: Paragraph 3. Statistical methods. (DOCX 132 kb)
Additional file 4: Table S1. Models for predicting arterial events only and venous events only. (DOCX 17 kb)
Additional file 5: Table S2. Spearman’s correlation coefficient (*p* value) between selected LA-related antibodies as well as selected “non-canonical” antibodies for the antiphospholipid syndrome. (DOCX 19 kb)
Additional file 6: Table S3. Distribution of selected hemostatic parameters according to treatment with oral anticoagulation status at baseline. (DOCX 17 kb)
Additional file 7: Table S4. Baseline characteristics of the study cohort according to prior history of pregnancy complications. (DOCX 25 kb)
Additional file 8
**Figure S1**. Cumulative incidence of thrombosis in the total study cohort. (DOCX 23 kb)
Additional file 9: Figure S2. Thrombotic risk according to baseline presence of diabetes (A), smoking (B), and a prolonged lupus-sensitive aPTT (C). (DOCX 36 kb)
Additional file 10: Figure S3. Thrombotic risk according to baseline presence of established APS. (DOCX 36 kb)
Additional file 11: Table S5. Multivariable models for thrombotic risk in LA patients adjusted for oral anticoagulation at baseline. (DOCX 17 kb)
Additional file 12: Paragraph 4. Sensitivity analyses for developing the point-based thrombotic risk scoring system. (DOCX 21 kb)
Additional file 13: Figure S4. Calibration bar graph of the proposed empirical risk stratification rule. (DOCX 22 kb)
