# Supplementary material for: Cardiovascular risk factors are major determinants of thrombotic risk in patients with the lupus anticoagulant
Source: BMC Med. 2017 Mar 10;15:54. doi: 10.1186/s12916-017-0807-7 (PMC5345189; doi:10.1186/s12916-017-0807-7)
Supplement: Additional file 1: — Paragraph 1. Study design and endpoint. (DOCX 85 kb) [file 12916_2017_807_MOESM1_ESM.docx]

**Supplementary Paragraph 1 – Study design and endpoint**

The Vienna Lupus Anticoagulant and Thrombosis Study (LATS) is an ongoing, single-center, biobank-based, prospective observational cohort study including adult patients that repeatedly test positive for the lupus anticoagulant (two positive tests at least 12 weeks apart) with or without a history of thrombosis or pregnancy complications. After written informed consent, blood is drawn for biobank storage, and a detailed medical history about comorbidities, prior thrombotic complications, and pregnancy outcomes is ascertained. Patients are then followed-up with repetitive clinical visits at our department every 6 months during the first 5 years, and once every year thereafter, until the occurrence of death or censoring due to LA negativity. At each visit, we record the onset of potential thrombotic complications, and draw blood for the biobank. The study does not interfere with routine clinical care for these patients. Specifically, decisions about the initiation of anticoagulation are at the discretion of the treating physicians. The primary endpoint of this study is a composite of symptomatic, objectively-confirmed arterial and/or venous thrombosis. All prospective events have to be established by objective diagnostic methods, such as coronary angiography for myocardial infarction, and every potential thrombotic event is adjudicated by a panel of experts in the field of cardiovascular medicine who work at our center.
