# Supplementary material for: Cardiovascular risk factors are major determinants of thrombotic risk in patients with the lupus anticoagulant
Source: BMC Med. 2017 Mar 10;15:54. doi: 10.1186/s12916-017-0807-7 (PMC5345189; doi:10.1186/s12916-017-0807-7)
Supplement: Additional file 2: — Paragraph 2. Sample preparation. (DOCX 60 kb) [file 12916_2017_807_MOESM2_ESM.docx]

**Supplementary Paragraph 2 – Sample preparation**

Venous blood samples were collected into trisodium citrate Vacuette tubes (9 parts of whole blood, 1 of trisodium citrate 3.8 %, Greiner Bio-One, Kremsmuenster, Austria) using a 21-gauge butterfly needle (Greiner Bio-One, Kremsmuenster, Austria). Samples were processed within three hours after venipuncture and centrifuged at 2,500g for 15 minutes at 15°C, followed by a second step of centrifugation of the harvested plasma under the same conditions to obtain platelet-poor plasma. For the determination of Annexin 5 resistance, aliquots were coded for blind analysis, snap-frozen and stored at −80°C until analyses were performed extramurally in series.
