# Supplementary material for: Cardiovascular risk factors are major determinants of thrombotic risk in patients with the lupus anticoagulant
Source: BMC Med. 2017 Mar 10;15:54. doi: 10.1186/s12916-017-0807-7 (PMC5345189; doi:10.1186/s12916-017-0807-7)
Supplement: Additional file 3: — Paragraph 3. Statistical methods. (DOCX 132 kb) [file 12916_2017_807_MOESM3_ESM.docx]

**Supplementary Paragraph 3 – Statistical methods**

All statistical analyses were performed using Stata (Windows version 13.0, Stata Corp. Houston, TX, USA). Continuous variables were reported as medians [25^th^ – 75^th^ percentile], and categorical variables by absolute frequencies and percentages. Correlations between two continuous variables, such as antibody titers, were explored with Spearman’s rank correlation coefficient. The association between categorical variables, such as between “triple-positivity” and the history of thrombotic complications, was assessed using uni- and multivariable logistic regression. Rank-sum tests, Kruskal-Wallis tests, as well as multiple linear regression was applied to compare means of continuous variables between two or more groups. The median follow-up time was estimated with the reverse Kaplan-Meier method according to Schemper and Smith. In order to eliminate information bias regarding the ascertainment of thrombotic outcomes at potentially different time points, we truncated the follow-up period on December 31^st^, 2014. Patients that became LA-negative during follow-up (n=11) were censored at the date of the first negative LA test. In all time-to-primary-endpoint analyses, we treated death-from-any-cause as a competing risk. The cumulative incidence of the primary endpoint was calculated with competing risk cumulative incidence estimators according to Marubini & Valsecchi. Differences in thrombosis incidence functions between two or more groups were investigated using Gray’s test. The association between potential risk factors and the cumulative incidence of thrombosis was modelled with uni- and multivariable proportional subdistribution hazards models according to Fine & Gray. The proportionality of subdistribution hazards assumption was assessed for each predictor by fitting an interaction between the predictor and the natural logarithm of follow-up time. For modeling the lupus-sensitive aPTT, we used the lupus-sensitive aPTT ratio, defined as the ratio of the lupus-sensitive aPTT of a patient divided by the mean of the lupus-sensitive aPTT in healthy controls at our department (Mean=34.09sec, SD=0.476). The lupus-sensitive aPTT ratio was chosen to increase external generalizability to other population where other reagents or analysis systems are in use. A backward selection algorithm (p for exclusion=0.10) including all four univariable predictors of thrombotic risk with p<0.10 (lupus-sensitive aPTT ratio (dichotomized into a binary variable at the 75^th^ percentile of the distribution (117.5 seconds)), diabetes, smoking, aCL IgM antibodies) was applied to construct a multivariable model for the prediction of thrombotic risk (thrombin generation parameters were not considered because of a considerable proportion of missing data). The algorithm selected the three variables diabetes, smoking, and a prolongued lupus-sensitive aPTT, and we constructed an empirical risk stratification rule by assigning two points for diabetes, and one point for each of the risk factors smoking and a prolonged lupus-sensitive aPTT. These points were chosen because the log hazard ratios for smoking and the prolonged lupus-sensitive aPTT were very close to each other, and the log hazard ratio for diabetes was close to double the log hazard ratio for the other two predictors. Hence, the chosen points are consistent with an *additive* effect of these predictors on the log hazard scale. Discrimination of the proposed stratification rule was assessed using Harell’s C statistic, and calibration was explored by comparing the observed and predicted 5- and 10-year cumulative incidences of thrombosis. Finally, in a sensitivity analysis, we assessed the separate association between the three risk stratification variables and the prospective risk of arterial and venous thrombosis.
