# Supplementary material for: Cardiovascular risk factors are major determinants of thrombotic risk in patients with the lupus anticoagulant
Source: BMC Med. 2017 Mar 10;15:54. doi: 10.1186/s12916-017-0807-7 (PMC5345189; doi:10.1186/s12916-017-0807-7)
Supplement: Additional file 4: Table S1. — Models for predicting arterial events only and venous events only. (DOCX 17 kb) [file 12916_2017_807_MOESM4_ESM.docx]

| **Table S1. Models for predicting arterial events only and venous events only** | | | | | | | | |
| --- | --- | --- | --- | --- | --- | --- | --- | --- |
|  | | | | | | | | |
|  |  | Univariable Models | | |  | Multivariable Model | | |
|  |  |  |  |  |  |  |  |  |
|  |  | **SHR** | **95%CI** | **p** |  | **SHR** | **95%CI** | **p** |
| **Models for all events** |  |  |  |  |  |  |  |  |
| Diabetes |  | 5.18 | 1.87-14.31 | 0.002 |  | 4.39 | 1.42-13.58 | 0.010 |
| Active Smoking |  | 2.11 | 1.06-4.20 | 0.034 |  | 2.39 | 1.14-5.02 | 0.021 |
| Prolonged aPTT-LA |  | 2.65 | 1.32-5.31 | 0.006 |  | 2.31 | 1.07-5.02 | 0.034 |
|  |  |  |  |  |  |  |  |  |
| **Models for arterial events** |  |  |  |  |  |  |  |  |
| Diabetes |  | 6.08 | 1.78-20.74 | 0.004 |  | 6.29 | 1.44-27.44 | 0.014 |
| Active Smoking |  | 2.24 | 0.85-5.89 | 0.104 |  | 2.65 | 0.92-7.62 | 0.071 |
| Prolonged aPTT-LA |  | 1.85 | 0.67-5.09 | 0.235 |  | 1.40 | 0.41-4.71 | 0.590 |
|  |  |  |  |  |  |  |  |  |
| **Models for venous events** |  |  |  |  |  |  |  |  |
| Diabetes |  | 2.38 | 0.53-10.67 | 0.257 |  | 2.04 | 0.49-8.46 | 0.327 |
| Active Smoking |  | 1.74 | 0.65-4.66 | 0.271 |  | 1.79 | 0.67-4.81 | 0.245 |
| Prolonged aPTT-LA |  | 3.15 | 1.20-8.31 | 0.020 |  | 3.01 | 1.16-7.84 | 0.024 |

All presented results are derived from uni- and multivariable Fine & Gray proportional subdistribution hazards regression models. Abbreviations: SHR – Subdistribution hazard ratio, 95%CI: 95% confidence interval, p – Wald test p-value, aPTT-LA – lupus-sensitive activated partial thromboplastin time.
