# Supplementary material for: Cardiovascular risk factors are major determinants of thrombotic risk in patients with the lupus anticoagulant
Source: BMC Med. 2017 Mar 10;15:54. doi: 10.1186/s12916-017-0807-7 (PMC5345189; doi:10.1186/s12916-017-0807-7)
Supplement: Additional file 5: Table S2. — Spearman’s correlation coefficient (p value) between selected LA-related antibodies as well as selected “non-canonical” antibodies for the antiphospholipid syndrome. (DOCX 19 kb) [file 12916_2017_807_MOESM5_ESM.docx]

| Table S2. Spearman’s correlation coefficient (p-value)  between selected LA-related antibodies | | | | | | | | | | |
| --- | --- | --- | --- | --- | --- | --- | --- | --- | --- | --- |
|  |  |  |  |  |  |  |  |  |  |  |
|  | aCL IgM | aCL IgG | aβ2-GPI IgM | aβ2-GPI IgG | aProthr IgM | aProthr IgG | aPrZ IgM | aPrZ IgG | A5R | D1-aβ2-GPI IgG |
| aCL IgM | 1.00 |  |  |  |  |  |  |  |  |  |
| aCL IgG | 0.19  (0.023) | 1.00 |  |  |  |  |  |  |  |  |
| aβ2-GPI IgM | 0.83  (<0.001) | 0.17  (0.043) | 1.00 |  |  |  |  |  |  |  |
| aβ2-GPI IgG | 0.06  (0.487) | 0.81  (<0.001) | 0.08  (0.339) | 1.00 |  |  |  |  |  |  |
| aProthr IgM | 0.47  (<0.0001) | 0.09  (0.317) | 0.52  (<0.001) | -0.02  (0.825) | 1.00 |  |  |  |  |  |
| aProthr IgG | 0.10  (0.237) | 0.30  (0.0004) | 0.13  (0.117) | 0.24  (0.004) | 0.24  (0.005) | 1.00 |  |  |  |  |
| aPrZ IgM | 0.30  (0.0004) | 0.16  (0.06) | 0.30  (0.0003) | 0.10  (0.224) | 0.46  (<0.001) | 0.05  (0.560) | 1.00 |  |  |  |
| aPrZ IgG | -0.08  (0.346) | 0.07  (0.398) | -0.06  (0.507) | 0.13  (0.136) | 0.14  (0.090) | 0.22  (0.009) | 0.06  (0.470) | 1.00 |  |  |
| A5R | -0.25  (0.003) | -0.65  (<0.001) | -0.29  (0.0005) | -0.57  (<0.001) | -0.12  (0.171) | -0.20  (0.020) | -0.17  (0.042) | -0.02  (0.842) | 1.00 |  |
| D1-aβ2-GPI IgG | -0.02  (0.858) | 0.74  (<0.001) | 0.07  (0.394) | 0.77  (<0.001) | 0.05  (0.560) | 0.29  (0.001) | 0.15  (0.076) | 0.06  (0.504) | -0.58  (<0.001) | 1.00 |

In each cell, the number on top represent Spearman’s correlation coefficient, whereas the number on the bottom of the cell in round brackets represents a p-value from a hypothesis test examining the null hypothesis that this correlation coefficient is zero. Abbreviations: aCL – anti Cardiolipin, aβ2-GPI – anti β2-glycoprotein 1, aProthr – anti Prothrombin, aPrZ – anti Protein Z, A5R- Annexin A5 anticoagulant ratio, D1-aβ2-GPI – antibodies against domain 1 of β2-glycoprotein 1, IgM – Immunoglobin M, IgG – Immunoglobin G.
