# Supplementary material for: Cardiovascular risk factors are major determinants of thrombotic risk in patients with the lupus anticoagulant
Source: BMC Med. 2017 Mar 10;15:54. doi: 10.1186/s12916-017-0807-7 (PMC5345189; doi:10.1186/s12916-017-0807-7)
Supplement: Additional file 6: Table S3. — Distribution of selected hemostatic parameters according to treatment with oral anticoagulation status at baseline. (DOCX 17 kb) [file 12916_2017_807_MOESM6_ESM.docx]

| Table S3. Distribution of selected haemostatic parameters according to treatment with oral anticoagulation status at baseline | | | | | | |
| --- | --- | --- | --- | --- | --- | --- |
|  |  |  |  |  |  |  |
| Variable |  | n (%miss.) | Overall  (n=150) | No VKA  at baseline  (n=80) | VKA  at baseline (n=70) | p |
|  |  |  |  |  |  |  |
| Fibrinogen (mg/dL) |  | 150 (0.0%) | 377 [318-430] | 368 [305-419] | 390 [322-447] | 0.111 |
| aPTT-LA (seconds) |  | 150 (0.0%) | 87.4 [70.1-117.5] | 79.7 [65.9-115.6] | 94.5 [74.5-118.1] | 0.086 |

Continuous variables are reported as medians [25^th^-75^th^ percentile], and categorical variables as absolute frequencies (%). Abbreviations: VKA – vitamin K antagonist, p – p-value from Wilcoxon’s rank-sum test.
