# Supplementary material for: Cardiovascular risk factors are major determinants of thrombotic risk in patients with the lupus anticoagulant
Source: BMC Med. 2017 Mar 10;15:54. doi: 10.1186/s12916-017-0807-7 (PMC5345189; doi:10.1186/s12916-017-0807-7)
Supplement: Additional file 7: Table S4. — Baseline characteristics of the study cohort according to prior history of pregnancy complications. (DOCX 25 kb) [file 12916_2017_807_MOESM7_ESM.docx]

| **Table S4. Baseline characteristics of the study cohort according to prior history of pregnancy complications** | | | | | | | |
| --- | --- | --- | --- | --- | --- | --- | --- |
|  |  |  |  |  |  |  |  |
| **Variable** | **N**  **(% miss.)** |  | **Overall**  **(n=94)** |  | **No pregnancy complications**  **(n=54)** | **≥1 pregnancy complication(s)**  **(n=40)** | **p** |
|  |  |  |  |  |  |  |  |
| **Demographic characteristics** |  |  |  |  |  |  |  |
| Age at entry (years) | 94 (0.0%) |  | 43.3 [35.8-60.3] |  | 54.8 [39.7-68.1] | 37.6 [32.9-45.9] | <0.0001 |
| BMI (kg/m²) | 94 (0.0%) |  | 25.9 [22.8-30.1] |  | 26.0 [23.3-29.8] | 25.6 [22.3-31.0] | 0.842 |
|  |  |  |  |  |  |  |  |
| **Clinical history** |  |  |  |  |  |  |  |
| Prior history of thrombosis | 94 (0.0%) |  | 61 (64.9%) |  | 34 (63.0%) | 27 (67.5%) | 0.649 |
| ---Arterial | 94 (0.0%) |  | 14 (14.9%) |  | 7 (13.0%) | 7 (17.5%) | 0.541 |
| ---Venous | 94 (0.0%) |  | 51 (54.3%) |  | 28 (51.9%) | 23 (57.5%) | 0.587 |
| Established APS | 94 (0.0%) |  | 74 (78.7%) |  | 34 (63.0%) | 40 (100.0%) | <0.0001 |
| Family history of thrombosis | 94 (0.0%) |  | 30 (31.9%) |  | 20 (37.0%) | 10 (25.0%) | 0.216 |
|  |  |  |  |  |  |  |  |
| **Comorbidities** |  |  |  |  |  |  |  |
| Hypertension | 94 (0.0%) |  | 31 (33.0%) |  | 20 (37.0%) | 11 (27.5%) | 0.331 |
| Diabetes | 94 (0.0%) |  | 6 (6.4%) |  | 3 (5.6%) | 3 (7.5%) | 0.703 |
| Autoimmune rheumatic diseases | 94 (0.0%) |  | 34 (36.2%) |  | 21 (38.9%) | 13 (32.5%) | 0.524 |
| Hereditary Thrombophilia* | 94 (0.0%) |  | 26 (27.7%) |  | 17 (31.5%) | 9 (22.5%) | 0.336 |
| Active smoker at baseline | 94 (0.0%) |  | 25 (26.6%) |  | 13 (24.1%) | 12 (30.0%) | 0.520 |
|  |  |  |  |  |  |  |  |
| **Anticoagulation at baseline** |  |  |  |  |  |  |  |
| VKA | 94 (0.0%) |  | 41 (43.6%) |  | 21 (38.9%) | 20 (50.0%) | 0.283 |
| LMWH | 94 (0.0%) |  | 13 (13.8%) |  | 5 (9.3%) | 8 (20.0%) | 0.136 |
| LDA | 94 (0.0%) |  | 26 (27.7%) |  | 15 (27.8%) | 11 (27.5%) | 0.976 |
| None | 94 (0.0%) |  | 33 (35.1%) |  | 21 (38.9%) | 12 (30.0%) | 0.372 |
|  |  |  |  |  |  |  |  |
| **Disease-defining autoantibodies** |  |  |  |  |  |  |  |
| aPTT-LA (sec) | 94 (0.0%) |  | 85.7 [66.5-116.1] |  | 80.6 [66.1-116.1] | 88.7 [69.9-121.0] | 0.499 |
| aβ2-GPI IgM (MPL) | 93 (1.1%) |  | 5.6 [2.5-15.6] |  | 5.0 [2.5-14.7] | 6.2 [2.8-17.2] | 0.463 |
| aβ2-GPI IgG (GPL) | 94 (0.0%) |  | 9.0 [2.1-50.8] |  | 4.4 [1.5-46.9] | 18.5 [5.0-67.2] | 0.012 |
| aCL IgM (MPL) | 94 (0.0%) |  | 9.1 [3.7-19.6] |  | 8.4 [3.3-16.2] | 9.2 [4.1-23.7] | 0.398 |
| aCL IgG (GPL) | 94 (0.0%) |  | 17.7 [6.0-80.5] |  | 11.4 [5.1-54.6] | 31.3 [10.6-104.7] | 0.030 |
| LA alone | 93 (1.1%) |  | 28 (30.1%) |  | 19 (35.9%) | 9 (22.5%) | 0.165 |
| LA + aβ2-GPI | 93 (1.1%) |  | 64 (68.9%) |  | 33 (62.3%) | 31 (77.5%) | 0.116 |
| LA + aCL | 94 (0.0%) |  | 38 (40.4%) |  | 18 (33.3%) | 20 (50.0%) | 0.104 |
| Triple positivity | 93 (1.1%) |  | 36 (38.7%) |  | 16 (30.2%) | 20 (50.0%) | 0.052 |
|  |  |  |  |  |  |  |  |
| **LA-related autoantibodies** |  |  |  |  |  |  |  |
| DI-aß2-GPI (CU) | 93 (1.1%) |  | 30.4 [2.0-201.0] |  | 5.1 [1.8-64.8] | 153.4 [15.7-265.5] | 0.005 |
| aPrZ IgM | 90 (4.3%) |  | 6.1 [4.3-9.6] |  | 5.8 [4.1-8.3] | 6.8 [4.5-10.8] | 0.129 |
| aPrZ IgG | 90 (4.3%) |  | 3.3 [2.5-5.4] |  | 3.3 [2.7-4.8] | 3.4 [2.3-6.2] | 0.929 |
| aProthr IgM | 91 (3.2%) |  | 5.1 [3.2-10.1] |  | 4.1 [3.2-8.5] | 6.4 [3.2-12.1] | 0.280 |
| aProthr IgG | 91 (3.2%) |  | 4.2 [2.0-8.4] |  | 4.0 [1.7-6.4] | 4.9 [2.6-11.9] | 0.162 |
| A5R (%) | 93 (1.1%) |  | 218 [179-255] |  | 232 [184-255] | 201 [174-252] | 0.251 |
|  |  |  |  |  |  |  |  |
| **Selected laboratory parameters** |  |  |  |  |  |  |  |
| C-reactive protein | 93 (1.1%) |  | 0.1 [0.0-0.7] |  | 0.1 [0.0-0.8] | 0.2 [0.0-0.6] | 0.952 |
| Triglycerides | 92 (2.1%) |  | 106 [82-146] |  | 104 [80-150] | 113 [82-145] | 0.834 |
| Cholesterol | 93 (1.1%) |  | 200 [174-229] |  | 199 [178-233] | 201 [169-226] | 0.737 |
| HDL/LDL ratio | 91 (3.2%) |  | 0.5 [0.4-0.7] |  | 0.5 [0.4-0.7] | 0.5 [0.4-0.6] | 0.926 |
| Homocystein | 88 (6.4%) |  | 9.8 [8.4-13.0] |  | 10.4 [8.3-14.5] | 9.6 [8.7-11.1] | 0.327 |
| Fibrinogen (mg/dL) | 94 (0.0%) |  | 379 [324-430] |  | 374 [324-459] | 385 [321-414] | 0.566 |
| Soluble P-selectin | 93 (1.1%) |  | 38.3 [26.0-51.8] |  | 36.6 [26.1-46.0] | 39.5 [25.7-54.3] | 0.452 |

Continuous variables are reported as medians [25^th^-75^th^ percentile], and categorical variables as absolute frequencies (%). Pregnancy complications were defined according to Sapporo criteria in the subgroup of 94 females that had at least one documented pregnancy. Autoimmune rheumatic diseases were defined as a composite of systemic lupus erythematosus (SLE) and lupus-like disease (LLD) according to a local panel of rheumatology experts. *Hereditary thrombophilia was defined as the presence of at least one of the following factors: (1) heterozygous or homozygous factor V Leiden, (2) deficiency of antithrombin III, (3) deficiency of protein C or protein S, (4) hyperhomocysteinemia, (5) heterozygous or homozygous prothrombin G20210A polymorphism, and (6) high coagulation factor VIII. P-values come from Wilcoxon’s rank-sum tests. Abbreviations: BMI – body mass index, APS – antiphospholipid syndrome, VKA – vitamin K antagonist, LMWH – low molecular weight heparin, LDA – low dose aspirin, aPTT-LA – lupus-sensitive activated partial thromboplastin time, aCL – anti Cardiolipin, aβ2-GPI – anti β2-glycoprotein 1, aProthr – anti Prothrombin, aPrZ – anti Protein Z, A5R- Annexin A5 anticoagulant ratio, D1-aβ2-GPI – antibodies against domain 1 of β2-glycoprotein 1, IgM – Immunoglobin M, IgG – Immunoglobin G.
