# Supplementary material for: Cardiovascular risk factors are major determinants of thrombotic risk in patients with the lupus anticoagulant
Source: BMC Med. 2017 Mar 10;15:54. doi: 10.1186/s12916-017-0807-7 (PMC5345189; doi:10.1186/s12916-017-0807-7)
Supplement: Additional file 8 — Figure S1. Cumulative incidence of thrombosis in the total study cohort. (DOCX 23 kb) [file 12916_2017_807_MOESM8_ESM.docx]

**Figure S1. Cumulative incidence of thrombosis in the total study cohort.** The cumulative incidence of thrombosis was estimated using competing risk cumulative incidence estimators that treat all-cause mortality as the competing event of interest.
