# Supplementary material for: Cardiovascular risk factors are major determinants of thrombotic risk in patients with the lupus anticoagulant
Source: BMC Med. 2017 Mar 10;15:54. doi: 10.1186/s12916-017-0807-7 (PMC5345189; doi:10.1186/s12916-017-0807-7)
Supplement: Additional file 10: Figure S3. — Thrombotic risk according to baseline presence of established APS. (DOCX 36 kb) [file 12916_2017_807_MOESM10_ESM.docx]

**Supplementary Figure 3**

**Figure S3. Thrombotic risk according to baseline presence of established APS.** The cumulative incidence of thrombosis was estimated using competing risk cumulative incidence estimators that treat all-cause mortality as the competing event of interest. Cumulative incidence functions between the two groups were compared using Gray’s test.
