# Supplementary material for: Cardiovascular risk factors are major determinants of thrombotic risk in patients with the lupus anticoagulant
Source: BMC Med. 2017 Mar 10;15:54. doi: 10.1186/s12916-017-0807-7 (PMC5345189; doi:10.1186/s12916-017-0807-7)
Supplement: Additional file 11: Table S5. — Multivariable models for thrombotic risk in LA patients adjusted for oral anticoagulation at baseline. (DOCX 17 kb) [file 12916_2017_807_MOESM11_ESM.docx]

| Table S5. Multivariable models for thrombotic risk in LA patients adjusted for oral anticoagulation at baseline | | | | |
| --- | --- | --- | --- | --- |
| Models |  | SHR | 95%CI | p |
|  |  |  |  |  |
| Model #1 |  |  |  |  |
| Diabetes |  | 4.11 | 1.32-12.81 | 0.015 |
| Active Smoking |  | 2.51 | 1.18-5.31 | 0.016 |
| Prolonged aPTT-LA ratio* |  | 2.28 | 1.04-4.97 | 0.039 |
| Oral anticoagulation at baseline (VKA) |  | 1.18 | 0.57-2.45 | 0.653 |
|  |  |  |  |  |
| Model #2 |  |  |  |  |
| 0 points (n= 77 (51.3%)) |  | Ref. | Ref. | Ref. |
| 1 point (n=51 (34.0%)) |  | 2.86 | 1.16-7.09 | 0.023 |
| ≥2 points (n=22 (14.7%)) |  | 8.84 | 3.25-24.03 | <0.0001 |
| Oral anticoagulation at baseline (VKA) |  | 1.18 | 0.57-2.46 | 0.655 |

Model #1 is a multivariable model including the three variables as binary specifications (a prolonged lupus-sensitive aPTT was defined as being above the 75^th^ percentile (Q3) of this variable’s distribution (cut-off: 117.5 seconds)). Model #2 is the final product of our prediction model building strategy, and observed risk according to this point-based rule are shown in Figure 1. Abbreviations: SHR – Subdistribution hazard ratio, 95%CI: 95% confidence interval, p – Wald test p-value, log(SHR) – Natural logarithm of the SHR, Ref. – Reference category, VKA – vitamin K antagonist. *prolonged aPTT ratio defined by a prolongation above the 75^th^ percentile of this variable’s distribution (this corresponds to cut-off at 117.5 seconds (or for the aPTT ratio at 3.4 multiples of the median in healthy individuals)).
