# Supplementary material for: Cardiovascular risk factors are major determinants of thrombotic risk in patients with the lupus anticoagulant
Source: BMC Med. 2017 Mar 10;15:54. doi: 10.1186/s12916-017-0807-7 (PMC5345189; doi:10.1186/s12916-017-0807-7)
Supplement: Additional file 12: — Paragraph 4. Sensitivity analyses for developing the point-based thrombotic risk scoring system. (DOCX 21 kb) [file 12916_2017_807_MOESM12_ESM.docx]

**Supplementary Paragraph 4 - Sensitivity analyses for developing the point-based thrombotic risk scoring system**

In this supplementary paragraph, we elaborate on a sensitivity analysis regarding the choice of our point-based thrombotic risk stratification rule.

The log(subdistribution hazard ratios) for active smoking and a prolonged lupus-sensitive aPTT ratio for prediction of thrombotic risk were 0.88 and 0.82, respectively (**Table 4, Model #1**). This similarity in relative contribution to thrombotic risk between active smoking and the prolonged lupus-sensitive aPTT ratio clearly suggested to us to assign **1 point** to these two variables.

The log(subdistribution hazard ratio) for diabetes was 1.38. Taking the arithmetic average of log(subhazard ratios) for active smoking and a prolonged lupus-sensitive aPTT ((0.88+0.82)/2=**0.85**), the relative contribution of diabetes towards thrombotic risk, as compared to these two variables, would be 1.38/0.85=**1.62**. We have rounded this relative contribution of 1.62 to **2 points**. Here, we perform a sensitivity analysis for the prognostic risk stratification rule **given we would have assigned 1 instead of 2 points for diabetes** (as suggested by a reviewer):

Assigning 1 instead of 2 points for diabetes yields the following alternative risk stratification variable with a 3-level point distribution:

| Freq. Percent Cum.

------------+-----------------------------------

0 | 77 51.33 51.33

1 | 53 35.33 86.67

2 | 20 13.33 100.00

------------+-----------------------------------

Total | 150 100.00

We now fit a multivariable competing risk regression model for thrombotic risk for this altnerative variable:

|  |  | **SHR** | **95%CI** | **p** |
| --- | --- | --- | --- | --- |
| **0 points (n=77 (51%)** |  | Ref. | Ref. | Ref. |
| **1 point (n=53 (35%)** |  | 3.25 | 1.34-7.83 | 0.009 |
| **2 points (n=20 (13%)** |  | 7.21 | 2.63-19.71 | <0.0001 |

This analysis demonstrates that this “**alternative”** score is also highly associated with thrombotic risk. **As compared to the “original” score, there is only a very negligible change in regression coefficients upon assigning 1 instead of 2 points for diabetes. In detail, the SHR for the 1 point category is somewhat higher (3.25 vs. 2.84) and somewhat stronger associated (p=0.009 vs. p=0.024) with thrombotic risk in the “alternative” score, whereas the SHR for the 2 point category is somewhat lower (7.21 vs. 8.53) and somewhat weaker associated (p=0.0001 vs. p=0.00002) with thrombotic risk in the “alternative” score.**

We now estimate the observed cumulative incidences of thrombotic risk according to the “altnerative” score variable. **In detail, the 10-year thrombotic risk was estimated 9.7%, 33.5%, and 52.5% in patients with 0 points, 1 point, and 2 points, respectively. This is extremely similar to the “original” risk stratification rule, which estimates 10-year thrombotic risk of 9.7%, 30.9%, and 56.8% in patients with 0 points, 1 point, and ≥ 2 points, respectively.**

**The thrombotic outcomes of our cohort according to the “alternative” score are plotted in the figure below:**

We then further evaluated the “alternative” and “original” score by comparing their Harrell’s C coefficients (the higher, the better the discrimation), their Akaike’s information criteria (AIC; the lower, the better the model’s fit) and their Bayesian information criteria (BIC; the lower, the better the model’s fit):

| **Model** |  | **Harrell’s C** | **AIC** | **BIC** |
| --- | --- | --- | --- | --- |
|  |  |  |  |  |
| **“Original score”** |  | 0.72 | 281.8 | 284.8 |
| **“Reviewer #3 score”** |  | 0.70 | 283.5 | 286.5 |

**This analysis shows that the “original score” has a somewhat higher discrimination (as indicated by Harrell’s C) and a somewhat better fit to the data (as indicated by the AIC and BIC) than the “altnerative” score. We therefore decided to keep the risk stratification in its original form, assigning 2 points for diabetes.**
