# Supplementary material for: Cardiovascular risk factors are major determinants of thrombotic risk in patients with the lupus anticoagulant
Source: BMC Med. 2017 Mar 10;15:54. doi: 10.1186/s12916-017-0807-7 (PMC5345189; doi:10.1186/s12916-017-0807-7)
Supplement: Additional file 13: Figure S4. — Calibration bar graph of the proposed empirical risk stratification rule. (DOCX 22 kb) [file 12916_2017_807_MOESM13_ESM.docx]

**Figure S4. Calibration bar graph of the proposed empirical risk stratification rule.** The bars represent predicted versus observed cumulative incidences of thrombosis. Predicted incidences were estimated from a Fine & Gray model including the point-based risk stratification rule, whereas observed cumulative incidence of thrombosis were estimated using non-parametric competing risk cumulative incidence estimators that treat all-cause mortality as the competing event of interest.
